# Mass spectrometric analysis of the in vitro secretome from equine bone marrow-derived mesenchymal stromal cells to assess the effect of chondrogenic differentiation on response to interleukin-1β treatment

**DOI:** 10.1186/s13287-020-01706-7

**Published:** 2020-05-20

**Authors:** Louise Bundgaard, Allan Stensballe, Kirstine Juul Elbæk, Lise Charlotte Berg

**Affiliations:** 1 Department of Veterinary Clinical Sciences, University of Copenhagen, Agrovej 8, 2630 Taastrup, Denmark; 2 Department of Health Science and Technology, Aalborg University, Fredrik Bajers Vej 7E, 9220 Aalborg Ø, Denmark

**Keywords:** Mesenchymal stromal cells, Equine, Mass spectrometry, Secretome, Inflammation, Chondrogenic differentiation, Joint disease

## Abstract

**Background:**

Similar to humans, the horse is a long-lived, athletic species. The use of mesenchymal stromal cells (MSCs) is a relatively new frontier, but has been used with promising results in treating joint diseases, e.g., osteoarthritis. It is believed that MSCs exert their main therapeutic effects through secreted trophic biomolecules. Therefore, it has been increasingly important to characterize the MSC secretome. It has been shown that the effect of the MSCs is strongly influenced by the environment in the host compartment, and it is a crucial issue when considering MSC therapy. The aim of this study was to investigate differences in the in vitro secreted protein profile between naïve and chondrogenic differentiating bone marrow-derived (BM)-MSCs when exposed to an inflammatory environment.

**Methods:**

Equine BM-MSCs were divided into a naïve group and a chondrogenic group. Cells were treated with normal expansion media or chondrogenic media. Cells were treated with IL-1β for a period of 5 days (stimulation), followed by 5 days without IL-1β (recovery). Media were collected after 48 h and 10 days. The secretomes were digested and analyzed by nanoLC-MS/MS to unravel the orchestration of proteins.

**Results:**

The inflammatory proteins IL6, CXCL1, CXCL6, CCL7, SEMA7A, SAA, and haptoglobin were identified in the secretome after 48 h from all cells stimulated with IL-1β. CXCL8, OSM, TGF-β1, the angiogenic proteins VCAM1, ICAM1, VEGFA, and VEGFC, the proteases MMP1 and MMP3, and the protease inhibitor TIMP3 were among the proteins only identified in the secretome after 48 h from cells cultured in normal expansion media. After 10-day incubation, the proteins CXCL1, CXCL6, and CCL7 were still identified in the secretome from BM-MSCs stimulated with IL-1β, but the essential inducer of inflammation, IL6, was only identified in the secretome from cells cultured in normal expansion media.

**Conclusion:**

The findings in this study indicate that naïve BM-MSCs have a more extensive inflammatory response at 48 h to stimulation with IL-1β compared to BM-MSCs undergoing chondrogenic differentiation. This extensive inflammatory response decreased after 5 days without IL-1β (day 10), but a difference in composition of the secretome between naïve and chondrogenic BM-MSCs was still evident.

## Background

Similar to humans, the horse is a long-lived, athletic species. Osteoarthritis (OA) including joint inflammation and cartilage degradation is one of the leading causes of lameness in horses, but there is still no cure to prevent the progressive degradation of the joint tissue or restore normal joint anatomy. Therefore, the goals for the treatments used for decades have been to slow the progression of the disease, minimize pain, and increase the function of the joint [1]. The use of mesenchymal stromal cells (MSCs) in treating joint diseases is a relatively new frontier, but has been used with promising results in the clinic [2, 3], and cell-based therapies are seen as the next-generation treatment of joint diseases.

It was originally proposed that transdifferentiation or cell fusion of transplanted MSCs was the principal mechanism underlying their therapeutic action in tissue regeneration [4]. But it is now believed that MSCs exert their main therapeutic effects through secreted trophic biomolecules acting in a paracrine fashion [5]. Therefore, it has been increasingly important to characterize the MSC secretome, and omics approaches have proved very useful for this purpose [6–9]. Indeed, identification of key MSC-secreted factors might be central in the design of next-generation joint disease therapeutics. The array of mechanisms affected by the MSC secretome spans from cell division and cell survival [10] to angiogenesis [11], extracellular matrix (ECM) organization [12], immunomodulation [13], and inflammatory response [14]. Previous studies have shown that the mode of MSC action is affected by the environment. A study of the secretome from human bone marrow (BM)-derived MSCs stimulated with IL-1β identified a broad spectrum of proteins involved in inflammation and angiogenesis which were overrepresented in the stimulated MSC secretome [9, 15], and another study of human osteoarthritic BM-MSCs undergoing chondrogenic differentiation reported a panel of extracellular markers potentially useful for cartilage repair after tissue engineering-based treatments [8]. These findings indicate that the effect of MSCs will be strongly influenced by the environment of the host compartment, and it is a crucial matter when considering MSC therapy.

The aim of this study was to investigate differences in the in vitro secreted proteome between naïve and chondrogenic differentiating BM-derived MSCs when exposed to an inflammatory (IL-1β) environment.

## Methods

### Study design

**Fig. 1:**
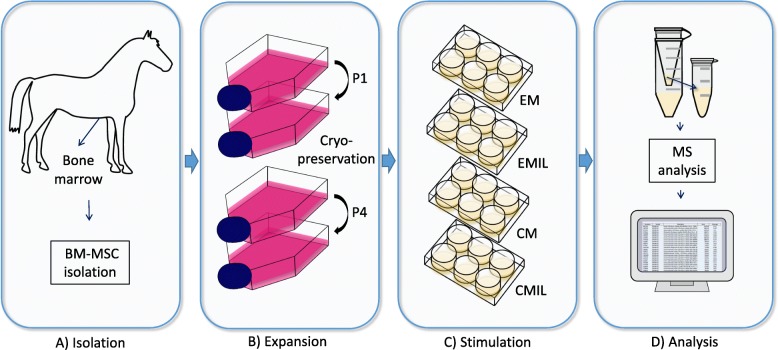
Schematic drawing of the workflow. **a** Bone marrow-derived mesenchymal stromal cells (BM-MSCs) were isolated from the equine sternal bone marrow. **b** BM-MSCs were expanded to P4. **c** The BM-MSCs were seeded in 6-well plates and divided in the following treatment groups: (1) expansion media without IL-1β (EM), (2) expansion media with IL-1β (EMIL) for 5 days followed by 5 days recovery time, (3) chondrogenic media without IL-1β (CM), and (4) chondrogenic media with IL-1β (CMIL) for 5 days followed by 5-day recovery time. Media in the 6 wells were harvested and pooled from each of the treatment groups after 48 h and 10 days. **d** Samples were concentrated in spin filters, processed, analyzed by mass spectrometry (MS), and the acquired data interpreted

Mesenchymal stromal cells derived from the bone marrow were divided into a naïve group and a chondrogenic group. Cells were treated with normal expansion media or chondrogenic media. Cells were treated with IL-1β for a period of 5 days (stimulation), followed by 5 days without IL-1β (recovery). Media were collected after 48 h and 10 days (Fig. 1).

### Isolation and expansion of mesenchymal stromal cells

BM-MSCs were obtained from a 15-year-old standard bred trotter mare. The horse was euthanized with captive bolt and exsanguination at the Large Animal Teaching Hospital, University of Copenhagen, for unrelated reasons.

The MSCs were isolated according to the procedure described in Bundgaard et al. [16]. In short, BM-MSCs were isolated from BM aspirated from the sternum, which together with tuber coxae is the most common location for bone marrow aspiration in the horse [17]. BM was aspirated using a Jamshidi biopsy needle (11 gauge, 12.7 cm) (Stryker, Kalamazoo, MI, USA) in a 20-mL syringe preloaded with 1 mL 10% 0.109 M trisodium citrate. The sample was further processed within ~ 1 h after sampling.

The mononuclear cell fraction was recovered after Histopaque-1077 (Sigma-Aldrich, St. Louis, MO, USA) separation, washed in sterile PBS, and resuspended in growth medium (GM) (Dulbecco’s modified Eagle’s medium (DMEM) 1 g/L glucose, with phenol red, GlutaMAX, and pyruvate (Thermo Fischer Scientific, Waltham, MA, USA) supplemented with 10% (v/v) fetal bovine serum (FBS) (Thermo Fischer Scientific, Waltham, MA, USA), 100 U/mL penicillin and 100 μg/mL streptomycin (Thermo Fischer Scientific, Waltham, MA, USA), and 25 μg/mL amphotericin B (Thermo Fischer Scientific, Waltham, MA, USA)). The cells were distributed equally in two T75 cm^2^ culture flasks and cultured at 37 °C in a humidified atmosphere containing 5% CO_2_. After overnight incubation, non-adherent cells were removed and fresh medium added. Medium was changed every 2–3 days. Cells were passaged at ~ 80% confluency (10–12 days) using trypsin-EDTA (0.25%) (Thermo Fischer Scientific, Waltham, MA, USA). P1 cells were further expanded in GM without amphotericin B. At 80% confluency (8–10 days), cells were detached with trypsin-EDTA, rinsed in sterile PBS, counted, and cryopreserved in FBS supplemented with 10% dimethyl sulfoxide (Sigma-Aldrich, St. Louis, MO, USA) in ~ 2 × 10^6^ aliquots. Cryopreserved MSCs (~ 2 × 10^6^ cells) were thawed and expanded in T75 cm^2^ flasks in GM without amphotericin B. At ~ 90% confluency (10–12 days), the cells were passaged as described and replated. When P4 cells reached ~ 90% confluency (5–6 days), they were harvested and validated for mesodermal differentiation capacity using protocols for osteogenesis and chondrogenesis.

### Interleukin-1β stimulation and chondrogenic differentiation

Cells (P5) were seeded in 6-well plates at a density of 50,000 cells/well (5208 cells/cm^2^) and expanded in GM without amphotericin B. Upon reaching ~ 80% confluency, wells were washed with sterile PBS and divided into four treatment groups (6 wells/group). The treatment groups include expansion media without IL-1β (EM), expansion media with IL-1β (EMIL) for 5 days followed by 5-day recovery time, chondrogenic media without IL-1β (CM), or chondrogenic media with IL-1β (CMIL) for 5 days followed by 5-day recovery time. All media were without phenol red, and to avoid interference from serum proteins in the results, FBS was substituted with insulin-transferrin-selenium (ITS) which has proven useful to sustain proliferation and differentiation in several cell types [18, 19]. EM consisted of DMEM 1 g/L glucose, supplemented with 1% (v/v) GlutaMAX (Thermo Fischer Scientific, Waltham, MA, USA), 1% (v/v) ITS (Thermo Fischer Scientific, Waltham, MA, USA), and 100 U/mL penicillin and 100 μg/mL streptomycin (Thermo Fischer Scientific, Waltham, MA, USA). CM consisted of DMEM 4.5 g/L glucose, supplemented with 1% (v/v) GlutaMAX (Thermo Fischer Scientific, Waltham, MA, USA), 1% (v/v) pyruvate, 1% (v/v) ITS, 100 U/mL penicillin and 100 μg/mL streptomycin, 10^−7^ M dexamethasone (Sigma-Aldrich, St. Louis, MO, USA), 50 μg/mL ascorbic acid (l-ascorbic acid 2-phosphate) (Sigma-Aldrich, St. Louis, MO, USA), and 10 ng/mL TGF-β3 (recombinant human TGF-β3) (R&D Systems, Inc., Minneapolis, MN, USA)). EMIL and CMIL were supplemented with IL-1β (1 μL/mL) (recombinant equine IL-1β) (R&D Systems, Inc., Minneapolis, MN, USA). A volume of 1-mL medium was added per well. After 48 h, media were harvested and pooled from each treatment group, centrifuged at 2000*g* for 2 min, snap frozen in liquid nitrogen, and stored at − 80 °C until further processing. Fresh treatment medium was added to each well including IL-1β for EMIL and CMIL. Medium was changed on days 5 and 8 to EM and CM without IL-1β for all wells. All media were harvested on day 10 and processed as described for medium harvested after 48 h.

### Cell differentiation into mesodermal lineages

Cells were passaged (P4) to 48-well plates at a density of ~ 10^3^ cells/well (9090 cells/cm^2^) and expanded in GM without amphotericin B. At ~ 80% confluency, cells were washed with sterile PBS and medium was changed to chondrogenic differentiation medium (DMEM 4.5 g/L glucose, without phenol red, supplemented with 1% (v/v) GlutaMAX (Thermo Fischer Scientific, Waltham, MA, USA), 1% (v/v) pyruvate (Thermo Fischer Scientific, Waltham, MA, USA), 2% (v/v) FBS, 1% (v/v) insulin-transferrin-selenium (ITS) (Thermo Fischer Scientific, Waltham, MA, USA), 100 U/mL penicillin and 100 μg/mL streptomycin, 10^−7^ M dexamethasone (Sigma-Aldrich, St. Louis, MO, USA), 50 μg/mL ascorbic acid (l-ascorbic acid 2-phosphate) (Sigma-Aldrich, St. Louis, MO, USA), 10 ng/mL TGF-β3 (recombinant human TGF-β3) (R&D Systems, Inc., Minneapolis, MN, USA)) or osteogenic differentiation medium (DMEM 1 g/L glucose, without phenol red, supplemented with 1% (v/v) pyruvate (Thermo Fischer Scientific, Waltham, MA, USA), 2% (v/v) FBS, 1% (v/v) ITS, 100 U/mL penicillin and 100 μg/mL streptomycin, 10^−7^ M dexamethasone, 50 μg/mL ascorbic acid (l-ascorbic acid 2-phosphate), 10 μL/mL 1 M β-glycerophosphate (β-glycerophosphate, disodium salt, pentahydrate) (Merck KGaA, Darmstadt, Germany)). Differentiation medium was changed every 3 days for 21 days. Confirmation of chondrogenic and osteogenic differentiation was performed by staining for proteoglycans in the extracellular matrix using 0.1% Safranin O, pH 4.6 (Merck KGaA, Darmstadt, Germany), and calcified extracellular matrix deposits using 2% Alizarin red staining, pH 4.2 (Sigma-Aldrich, St. Louis, MO, USA), respectively.

### Processing of the conditioned medium

Samples were thawed on ice and the proteins concentrated by use of a 3-kDa cut-off spin filter (Amicon Ultra-4 Centrifugal filter unit) (Merck Millipore, Burlington, MA, USA) followed by acetone precipitation (6× acetone (v/v), 1 h, − 20 °C), which is the preferred preparation method to be free of salts and other disturbing agents [20]. The proteins were resuspended in 0.5 mL 0.5% sodium-deoxycylate (SDC) (Sigma-Aldrich, St. Louis, MO, USA) in 50 mM triethylammonium bicarbonate (TEAB) (Sigma-Aldrich, St. Louis, MO, USA) and the total protein measured on a spectrophotometer (DeNovix DS 11Fx) (Denovix, Wilmington, DE, USA) in duplicates. A volume corresponding to 200 μg protein was transferred to a 10-kDa cut-off spin filter (Amicon Ultra-0.5 Centrifugal filter unit) (Merck Millipore, Burlington, MA, USA) and 100 μL 0.5% SDC in 50 mM TEAB buffer was added. The proteins were reduced with 1:50 (v/v) 0.5 M tris(2-carboxyethyl)phosphine (Thermo Scientific, Waltham, MA, USA), alkylated with 1:10 (v/v) 0.5 M chloroacetamide (Sigma-Aldrich, St. Louis, MO, USA), and incubated 30 min at 37 °C followed by centrifugation for 15 min at 14,000*g*. The buffer was exchanged with 200 μL 0.5% SDC in 50 mM TEAB followed by centrifugation for 15 min at 14,000*g*, and the flow-through was discarded. Trypsin (1 μg/100 μg protein) was added, and the proteins were digested overnight at 37 °C. The digested samples were centrifuged 15 min at 14,000*g*, the filter washed by centrifugation with 100 μL 50 mM TEAB for 15 min at 14,000*g*, and the flow-through used for further processing.

The SDC was removed from the sample with phase extraction by the use of 3:1 (v/v) ethyl acetate and acidification by trifluoroacetic acid (pH < 2). After thorough vortexing and centrifugation for 1 min at 14,000*g*, the upper phase was discarded and the lower phase containing the digested proteins was dried down by vacuum centrifugation. The samples were stored at − 20 °C.

### LC-MS/MS analysis

Peptides were resuspended in resuspension buffer (2% acetonitrile, 0.1% trifluoracetic acid (Thermo Fischer Scientific, Waltham, MA, USA), 0.1% formic acid in MilliQ water), and a volume corresponding to ~ 1 μg peptide was analyzed by nanoLC-MS/MS (Thermo Scientific Dionex Ultimate 3000 RSLC) coupled in line to a Thermo Scientific Q Exactive HF mass spectrometer. The peptide separation was accomplished using a precolumn setup (Acclaim PepMap 100 C18 2 cm 100 μm precolumn; 75 μm 75 cm main column) (Thermo Fischer Scientific, Waltham, MA, USA) and a 60-min gradient from 10% buffer B (99.9% acetonitrile) to 35% buffer B and the buffer A being MilliQ water with 0.1% formic acid. The mass spectrometer was set to acquire MS1 data from *m*/*z* 375–1500 at *R* = 60k and MS2 at *R* = 30k allowing up to 20 precursor ions per MS1 scan.

### Data analysis

Raw data was searched against the *Equus caballus* reference sequence database from Uniprot (UP000002281; May 16, 2017; 22,698 proteins) using MaxQuant search engines (MaxQuant v.1.6.0.1 and Perseus v.1.6.0.2). Only proteins with at least two unique peptide sequences, FDR < 1%, and identified in all technical replicates were included. Label-free quantification (LFQ) was based on total ion chromatogram normalization [21]. A difference in intensity of the same protein in the different conditions > 3× was considered significant. The R statistical software version 3.3.2 (https://www.r-project.org) was used for further analysis of the data. The heatmap was generated in R by the use of R-packages “gplots” (https://CRAN.R-project.org/package=gplots) and “RColorBrewer” (https://CRAN.R-project.org/package=RColorBrewer). Protein descriptions and GO biological processes found in Uniprot were used to group identified proteins, and the online database STRING-DB vs 10.5 was used to analyze interactions between clusters of proteins [22]. The MS proteomics data have been deposited and made publicly available to the ProteomeXchange Consortium via the PRIDE partner repository with the dataset identifier PXD016842 [23].

## Results

### Cellular morphology and differentiation into mesodermal linages

Cells were plastic adherent and exhibited a fibroblast-like morphology. Chondrogenic differentiated cells stained positive for proteoglycans in the extracellular matrix and osteogenic differentiated cells stained positive for calcified extracellular matrix deposits on day 21 after induction of differentiation.

### Mass spectrometry analysis

A total of 706 proteins with at least 2 unique peptides were identified with a 5% FDR on the protein level. A scatter plot with log2-transformed LFQ values showed a Pearson correlation of > 0.97 for triplicate analysis (Supplementary 1 and 2).

**Fig. 2:**
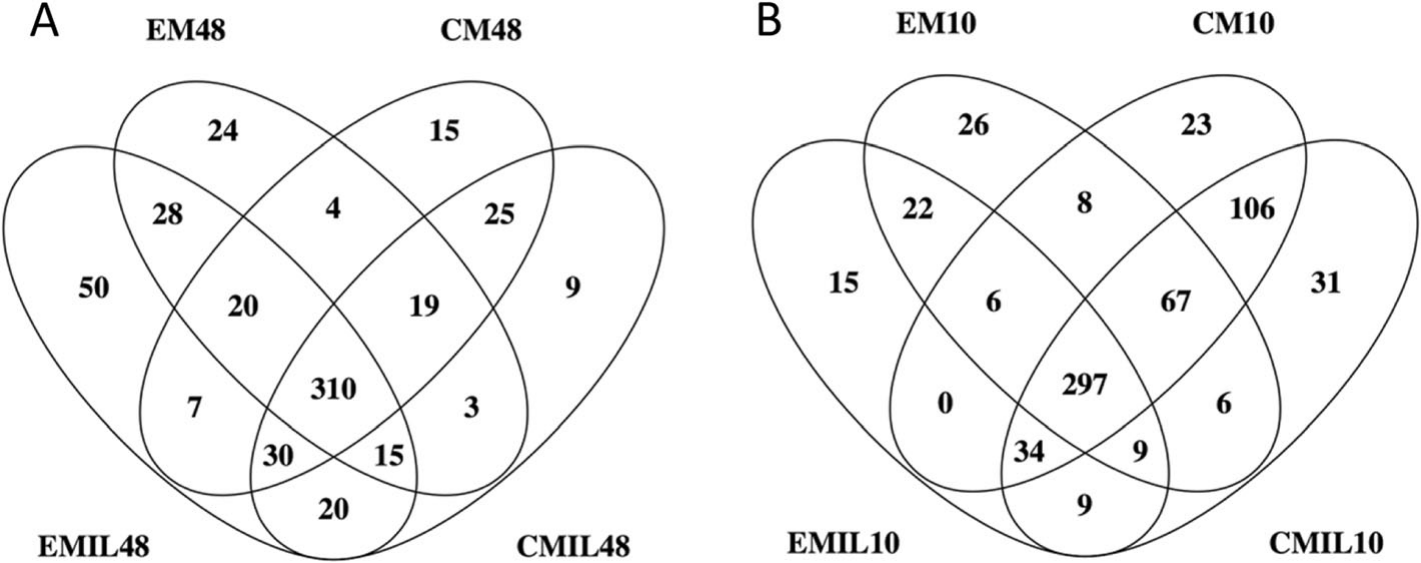
Secretomes harvested after 48 h and 10 days from BM-MSCs with or without IL-1β stimulation. Venn diagram comparing the secretomes harvested after **a** 48 h (48) and **b** 10 days (10) from equine bone marrow-derived mesenchymal stromal cells subjected to IL-1β stimulation (IL) for 5 days followed by 5 days without inflammation. At both time points, the secretome from naïve cells (EM) and chondrogenic differentiating cells (CM) was assessed

Figure 2a shows a Venn diagram comparing the secretomes from the four conditions after 48 h. A total of 309 proteins were common for all conditions. The proteins identified in the secretomes from the IL1β-stimulated conditions (EMIL48 and CMIL48) included a number of proteins involved in the acute inflammatory response such as IL1β, IL6, CXCL1, CXCL6, CCL7, semaphorin-7A (SEMA7A), the acute phase proteins serum amyloid A (SAA) and haptoglobin (HP), and the proteases MMP8 and antileukoproteinase (SLPI). Proteins identified only in the secretome from EMIL48 included the proteases MMP1 and MMP3 and the protease inhibitor TIMP3, as well as CXCL8 and oncostatin M (OSM), involved in the acute inflammatory response. Also, the angiogenic proteins VCAM1, ICAM1, VEGFA, and VEGFC were only identified in EMIL48. In the secretome from chondrogenic differentiated cells, a number of proteins involved in chondrogenesis were identified such as cartilage oligomeric matrix protein (COMP), stimulator of chondrogenesis 1 (SCRG1), xylotransferase 1 (XYLT1), and growth arrest-specific 6 (GAS6). Proteins identified only in the secretome from CMIL48 included vanin 1 (VNN1) and secreted phosphoprotein 1 (SPP1), also known as osteopontin, which both have a role in inflammatory diseases.

The Venn diagram comparing the secretomes from the four conditions after 10 h (Fig. 2b) showed a total of 297 proteins in common for all four conditions. Among the 9 proteins identified in both conditions stimulated with IL-1ß (EMIL10 and CMIL10) were the proteins CXCL1, CXCL6, CCL7, MMP1, and MMP19. IL6 was only identified in EMIL10. Secretogranin III (SCG3), involved in platelet degranulation and angiogenesis, and cartilage acidic protein 1 (CRTAC1), a protein upregulated in synovial fluid from humans with OA, were also only identified in the secretome from EMIL10. Proteins identified only in the secretome from CMIL10 included the inflammatory proteins SAA and IL11, gremlin (GREM1) involved in regulation of chondrocyte and osteoblast proliferation, and proteins involved in ECM organization, e.g., laminin subunit gamma-2 (LAMC2), cathepsin S (CTSS), and α-2-macroglobulin like 1 (A2ML1). In the secretome from chondrogenic differentiated cells (CM10 and CMIL10), a number of proteins involved in cartilage formation and bone mineralization were identified such as COMP, SCRG1, epiphycan (EPYC), osteomodulin (OMD), asporin (ASPN), and alkaline phosphatase (ALPL).

**Fig. 3:**
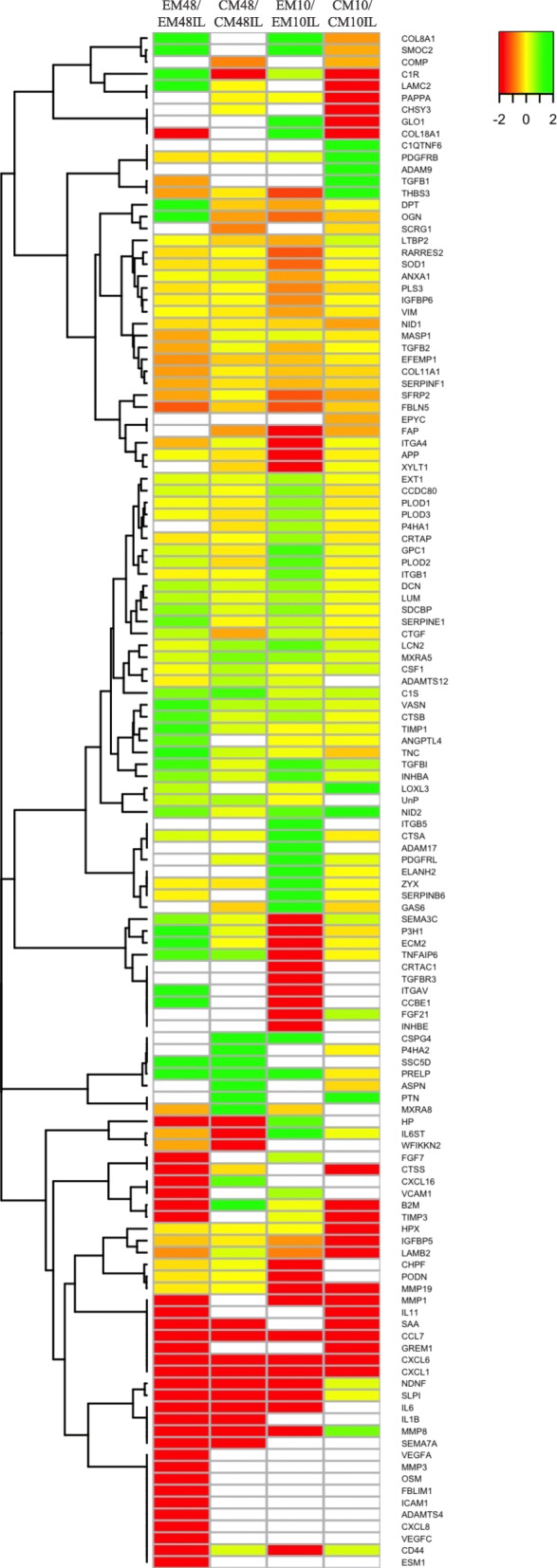
Differences in the secretomes harvested after 48 h and 10 days from BM-MSCs with or without IL-1β stimulation. Heatmap showing the difference in protein intensity in the secretomes harvested after 48 h (48) and 10 days (10) from equine bone marrow-derived mesenchymal stromal cells subjected to IL-1β stimulation (IL) for 5 days followed by 5 days without inflammation. At both time points, the secretome from naïve cells (EM) and chondrogenic differentiating cells (CM) was assessed. The proteins included are involved in either the inflammatory response, extracellular matrix organization, or chondrogenesis. The data has been log10 transformed

Figure 3 presents the majority of identified proteins involved in either the inflammatory response, ECM organization, or chondrogenesis in a heatmap based on the difference in intensity between EM48/EMIL48, CM48/CMIL48, EM10/EMIL10, and CM10/CMIL10 respectively. With a focus on the proteins differing in intensity between the different conditions after 48 h, it was found that TGF-β1 was identified with a higher intensity in the secretome from EM48IL compared to EM48. The intensity of the protein interleukin 6 receptor beta unit (IL6ST), important in the receptor system for various inflammatory proteins and also osteoblast differentiation, was significantly higher in the secretome from inflammatory-stimulated cells compared to non-stimulated.

Analysis of the intensity of proteins after 10 days showed that amyloid-beta precursor protein (APP), prolyl endopeptidase FAP (FAP), and integrin alpha-4 (ITGA4) involved in various processes including both ECM organization and the inflammatory response were identified in the secretome from naïve cells only after stimulation with IL-1β, while they were identified in the secretome from both CM10 and CMIL10, and only FAP had a significantly higher intensity in the secretome from CM10IL compared to CM10. Cartilage acidic protein 1 (CRTAC1) was only identified in EMIL10. The enzyme (XYLT1) involved in cartilage ECM organization was identified in both CM10 and CMIL10, but only in the secretome from EMIL10.

The cell surface receptor CD44, which is a mesenchymal stromal cell marker, but also a receptor that participates in a wide variety of cellular functions including the inflammatory response and ECM organization, was identified with a significantly higher intensity in the secretome from EMIL compared to secretome from non-stimulated naïve cells both after 48 h and 10 days. The intensity of CD44 in the secretome from CM and CMIL did not differ significantly at the two time points.

The proteins lumican and decorin expressed in cartilage were identified with the same intensity in the secretome from chondrogenic differentiated cells after 48 h and 10 days regardless of IL-1β stimulation. The intensity for these two proteins was higher in the secretome from non-stimulated naive cells compared to IL-1β-stimulated cells after both 48 h and 10 days, but it was not significant. Connective tissue growth factor (CTGF), a protein involved in chondrogenesis, was observed in a significantly higher intensity in IL-1β stimulated compared to non-stimulated chondrogenic cells after 48 h, but had the same intensity after 10 days. CTGF was also expressed in EM and EMIL after both 48 h and 10 days, but with no significant difference. The intensity for procollagen-lysine,2-oxoglutarate 5 (PLOD) 1, PLOD 2, PLOD 3, and cartilage associated protein (CRTAP), important proteins for collagen fibril formation, was in the same range in the secretome from all chondrogenic differentiated cells after 48 h and 10 days, and from EM48, but had a tendency towards a lower intensity in the secretome from EMIL10 compared to EM10. EFEMP1, a negative regulator of chondrogenesis, was identified in a higher concentration in the secretome for EMIL. The concentration did not differ in chondrogenic differentiating cells.

## Discussion

In this study, BM-MSCs were subjected to inflammation for 5 days followed by 5 days without inflammation. Samples were harvested after 48 h of inflammation mimicking acute inflammation [24, 25], and again on day 10 to evaluate the cells’ recovery after the inflammatory insult. At both time points, the modulatory effect of chondrogenic differentiation on the inflammatory response was assessed.

The findings in this study indicate that naïve BM-MSCs have a more extensive inflammatory response at 48 h to stimulation with IL-1β compared to BM-MSCs undergoing chondrogenic differentiation (EMIL48 and CMIL48). This extensive inflammatory response decreased after 5 days without IL-1β (day 10), but a difference in composition of the secretome between naïve and chondrogenic BM-MSC (EMIL10 and CMIL10) was still evident.

It is well known that IL-1β activates the nuclear factor-kB (NF-kB) pathway, a proinflammatory signaling pathway leading to release of a large array of cytokines (e.g., IL1, IL6, CXCL8), chemokines (e.g., CXCL1, CXCL10, IL18), adhesion molecules (e.g., ICAM1, VCAM1, MMPs), and various other proteins involved in inflammation [26]. The inflammatory proteins IL6, CXCL1, CXCL6, CCL7, SEMA7A, SAA, and haptoglobin were all identified in the secretome after 48 h from cells stimulated with IL-1β. IL6 is known as a main downstream target of IL-1β via the NF-kB pathway [26], and IL6 is one of the most important inflammatory cytokines [27]. IL6 is a strong activator of the acute phase response with expression of SAA and HP among others [27]. The chemokines CXCL1 and CXCL6 are chemotactic cytokines involved in various pathological processes including inflammatory diseases, and it has been shown that both CXCL1, CXCL6, and SEMA7A are involved in the inflammatory response in joint-related diseases such as rheumatoid arthritis [28, 29]. Also, IL-1β was identified in the secretome from both EM48IL and CM48IL, but it was not possible to distinguish between IL-1β produced by BM-MSC and the recombinant equine IL-1β used for stimulation. In a previous study, the gene expression of IL-1β was upregulated in equine BM-MSCs after 24 h conditioning with IL-1β [24], which supports a positive feedback loop of IL-1β in equine BM-MSCs.

A number of proteins produced in response to NF-kB pathway activation were identified only in the secretome from EMIL48. CXCL8 and OSM are essential proinflammatory proteins similar to IL6. CXCL8 is released upon NF-kB pathway activation and is especially chemoattractant on neutrophils [30], and OSM in combination with IL-1 has been shown to upregulate various inflammatory and MMP genes [31]. CXCL8, OSM, and the proteases MMP1 and MMP3 and the protease inhibitor TIMP3 were among the proteins involved in the acute inflammatory response only identified in the secretome from EMIL48. Proinflammatory cytokines such as IL1β and IL6 have been shown to activate the mitogen-activated protein kinase and Janus kinase/signal transducers and activators of transcription (JAK/STAT) pathway leading to increased synthesis of MMP1 and MMP3 [32]. These proteases are among the MMPs playing a critical role in the destruction of articular cartilage in various joint diseases [32]. Upregulation of MMP1, MMP3, and the protease inhibitor TIMP3 in the secretome from EMIL48 suggests that naïve BM-MSCs promote proteolytic activity in response to IL-1β stimulation. The multifunctional cytokine TGF-β1 was also among the proteins with a significant higher concentration in the secretome from EMIL48 compared to EM48. In OA, it has been found that TGF-β expression increases in the early stages in an attempt to counteract the catabolic effects of inflammatory cytokines such as IL-1β or TNFα [33]. Also, the angiogenic proteins VCAM1, ICAM1, VEGFA, and VEGFC were only identified in EMIL48. The inflammatory response depends on migration of leukocytes, and VCAM1 and ICAM1 have been shown to be essential in the process of leukocyte emigration to sites of inflammation [34], and it is known that the angiogenic process on sites of inflammation is perpetuated primarily by VEGF [35]. This indicates that naive BM-MSCs stimulated with IL-1β also have angiogenic properties. The cellular response for naïve BM-MSCs to inflammatory stimulation is in line with the findings in a study of the secretome from human BM-MSCs stimulated with IL-1β, IL6, and TNFα. Here they also found an increase of a broad range of proteins related to inflammation, protease activity, and angiogenesis [9]. Gene expression of proinflammatory molecules in equine BM-MSCs was also increased after inflammatory conditioning for 12–72 h, and it was demonstrated that the inflammatory response profile was affected by the type of conditioning, concentration of the proinflammatory cytokines, and the duration of the stimulation [24, 25, 36].

In a previous study, it was shown that IL-1β inhibited chondrogenic differentiation of human BM-MSCs aspirated from femurs of patients undergoing total hip replacement [37]. They did not include non-differentiating cells, and cells were only analyzed after 28-day culture, but the findings highlight the importance of further studies on the effect of inflammation on MSC and developing strategies to prepare the MSCs for an inflammatory in situ environment. In our study, the two proteins VNN1 and SPP1 were identified in the secretome from CMIL48. VNN1 has been studied in many different inflammatory diseases showing that it has either a protective or a sensitizing role [38], but the role in joint diseases has not been studied. For SPP1, also known as osteopontin, it has been indicated that it causes a decrease of collagen 2 and COMP in chondrocytes from osteoarthritis [39], but in OA progression, it plays an important role as a regulator [40], and it has been identified as a susceptibility gene for RA [41].

Taken together, these data propose that naïve BM-MSCs have a more extensive inflammatory response, including proteolytic and angiogenic activity, to stimulation with IL-1β after 48 h (EMIL48) compared to BM-MSC undergoing chondrogenic differentiation (CMIL48). This indicates that chondrogenic differentiation or a chondrogenic environment has a protective mechanism to IL-1β stimulation.

After 10-day incubation, the proteins CXCL1, CXCL6, and CCL7 were still identified in the secretome from BM-MSC stimulated with IL-1β, as after 48-h incubation, but the essential inducer of the acute inflammatory response, IL6, was only identified in the secretome from EMIL10 and not CMIL10. This further supports that IL-1β induces inflammation in BM-MSCs and that chondrogenic differentiation or a chondrogenic environment works as a moderator to IL-1β stimulation and facilitates a faster recovery after IL-1β stimulation. Also, the proteases MMP1 and MMP19 were both identified in the secretome from EMIL10 and CMIL10 indicating that there was still some degradation activity [32]. The proteins SAA and IL11 that both induce angiogenesis in RA [42, 43] were identified in the secretome from CMIL10 only. This indicates that there still was some inflammatory induced angiogenesis activity in this condition.

It has been demonstrated that the secretome from human BM-MSC can be used to describe the molecular mechanism during chondrogenesis [8]. In this study, a number of proteins related to cartilage were identified in the secretome from BM-MSCs cultured in a chondrogenic environment validating that the cells were undergoing chondrogenesis. After 48 h, these proteins included COMP, an important protein in cartilaginous ECM [44], SCRG1 [45], XYLT1 [46], and GAS6. After 10 days, these proteins included COMP, SCRG1, EPYC [47], OMD [48], ASPN [49], and ALPL [50]. The proteins lumican and decorin are expressed in the cartilage [51] and have both been suggested as chondrogenic markers [52, 53]. They were identified in all conditions, but the MS spectral intensity profiles were markedly higher for these proteins in the chondrogenic conditions compared to the naïve conditions.

The composition of the secretome was analyzed by MS, and the raw data was searched against the *Equus caballus* reference database from Uniprot. In accordance with the guidelines from the Human Proteome Organization (HUPO) [54], only proteins with at least two unique peptide sequences were included in the analyses of the secretomes. It should be noted that the reference database does not yet cover the complete equine genome. Moreover, a substantial part of the sequences in the reference database are derived from transcripts, which cause redundancy in protein assignments and fragment variants of the same sequence. To minimize the impact of this fact on the results and further improve the validity and quality of the study outcome, we only included proteins identified in all three replicates in the final analyses of the secretomes.

In conclusion, this study showed that naïve BM-MSCs had a more extensive inflammatory response at 48 h to stimulation with IL-1β compared to BM-MSCs undergoing chondrogenic differentiation. This extensive inflammatory response decreased after 5-day recovery time without IL-1β (day 10), but a difference in composition of the secretome between naïve and chondrogenic BM-MSCs remained evident. This study is descriptive and shows the effect of differentiation status on sensitivity to inflammation in BM-MSCs. The work in this study is important when considering BM-MSC therapy, because the findings indicate that BM-MSCs will be strongly influenced by an inflammatory environment present in the host compartment upon injection even later after the inflammation has subsided and that chondrogenic differentiation or a chondrogenic environment may have a protective mechanism to IL-1β stimulation. Further studies are needed to evaluate if this effect is transferable to other MSC types and to establish the functional and mechanistic consequences for use of MSC in therapy.

## Supplementary information

**Additional file 1: Supplementary 1 and 2.** Scatter plot with log2-transformed label free quantification (LFQ) values from mass spectrometry analysis of the secretomes harvested after 1) 48 h (48) and 2) 10 days (10) from equine bone marrow-derived mesenchymal stromal cells subjected to IL-1β stimulation (IL) for five days followed by five days without inflammation. At both time points, the secretome from naïve cells (EM) and chondrogenic differentiating cells (CM) was assessed.

**Additional file 2: Supplementary 3.** Table with data used for the heatmap.

## Data Availability

The mass spectrometry proteomics data generated during the current study are available in the ProteomeXchange Consortium via the PRIDE partner repository with the dataset identifier PXD016842.

## Abbreviations

A2ML1: α-2-Macroglobulin like 1
ALPL: Alkaline phosphatase
APP: Amyloid-beta precursor protein
ASPN: Asporin
BM: Bone marrow
CM: Chondrogenic media
CMIL: Chondrogenic media with IL-1β
COMP: Cartilage oligomeric matrix protein
CRTAC1: Cartilage acidic protein 1
CRTAP: Cartilage-associated protein
CTGF: Connective tissue growth factor
CTSS: Cathepsin S
DMEM: Dulbecco’s modified Eagle’s medium
ECM: Extracellular matrix
EM: Expansion media
EMIL: Expansion media with IL-1β
EPYC: Epiphycan
FAP: Prolyl endopeptidase FAP
FBS: Fetal bovine serum
GAS6: Growth arrest-specific 6
GM: Growth medium
GREM1: Gremlin
HP: Haptoglobin
IL: Interleukin
IL6ST: Interleukin 6 receptor beta unit
ITGA4: Integrin alpha-4
ITS: Insulin-transferrin-selenium
JAK/STAT: Janus kinase/signal transducers and activators of transcription
LAMC2: Laminin subunit gamma-2
MMP: Matrix metalloproteinase
MSCs: Mesenchymal stromal cells
NF-kB: Nuclear factor-kB
OA: Osteoarthritis
OMD: Osteomodulin
OSM: Oncostatin M
PLOD: Procollagen-lysine,2-oxoglutarate 5
SAA: Serum amyloid A
SCG3: Secretogranin III
SCRG1: Stimulator of chondrogenesis 1
SDC: Sodium-deoxycylate
SEMA7A: Semaphorin-7A
SLPI: Antileukoproteinase
SPP1: Secreted phosphoprotein 1
TEAB: Triethylammonium bicarbonate
VNN1: Vanin 1
XYLT1: Xylotransferase 1
