# Supplementary figures and images for: Mass spectrometric analysis of the in vitro secretome from equine bone marrow-derived mesenchymal stromal cells to assess the effect of chondrogenic differentiation on response to interleukin-1β treatment
Source: Stem Cell Res Ther. 2020 May 20;11:187. doi: 10.1186/s13287-020-01706-7 (PMC7238576; doi:10.1186/s13287-020-01706-7)

## Slide 1
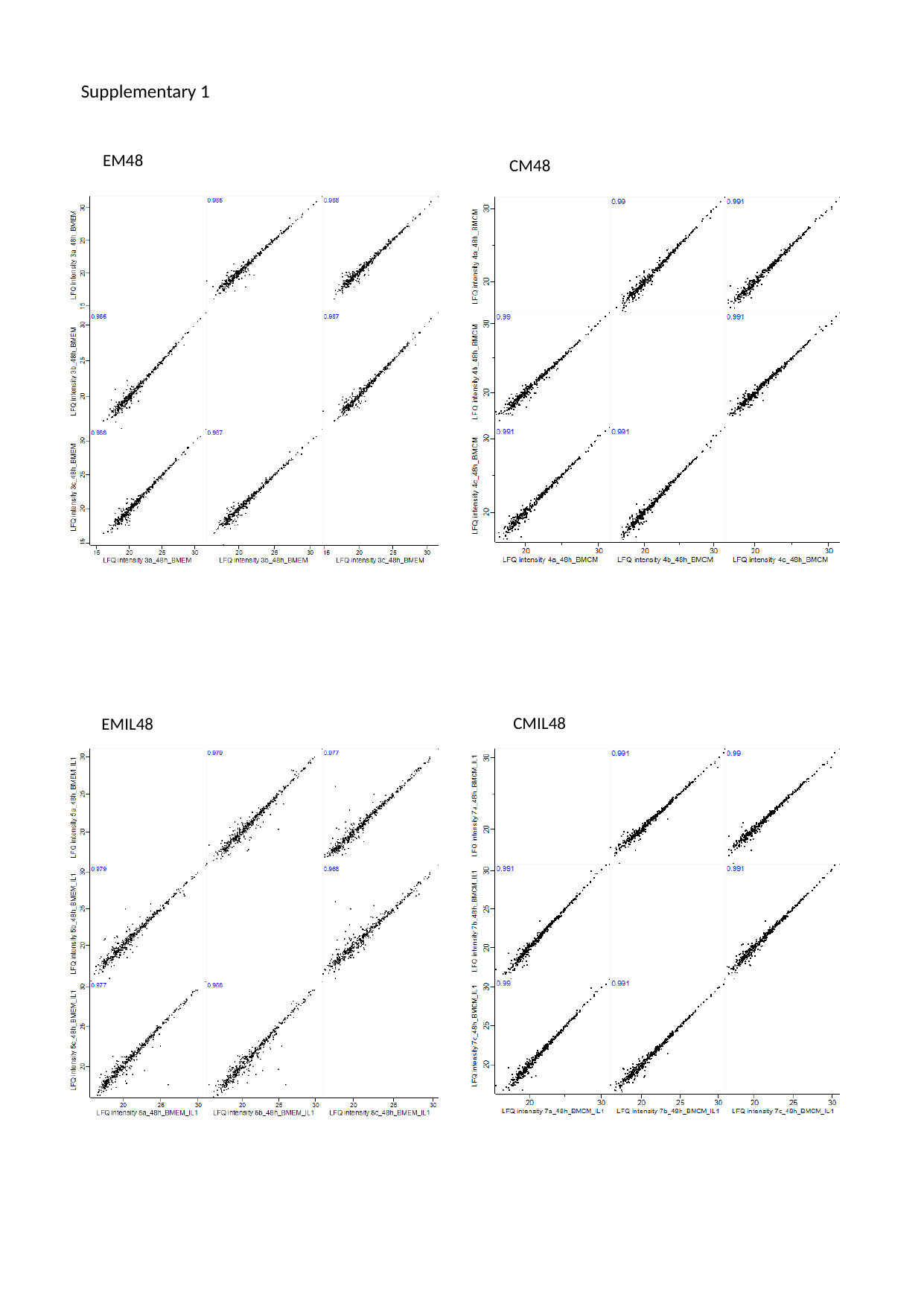

Supplementary 1
EM48
CM48
CMIL48
EMIL48

Supplement: Supplementary file 1 — Additional file 1: Supplementary 1 and 2. Scatter plot with log2-transformed label free quantification (LFQ) values from mass spectrometry analysis of the secretomes harvested after 1) 48 h (48) and 2) 10 days (10) from equine bone marrow-derived mesenchymal stromal cells subjected to IL-1β stimulation (IL) for five days followed by five days without inflammation. At both time points, the secretome from naïve cells (EM) and chondrogenic differentiating cells (CM) was assessed. [file 13287_2020_1706_MOESM1_ESM.zip › Supp_1.pptx]

## Slide 1
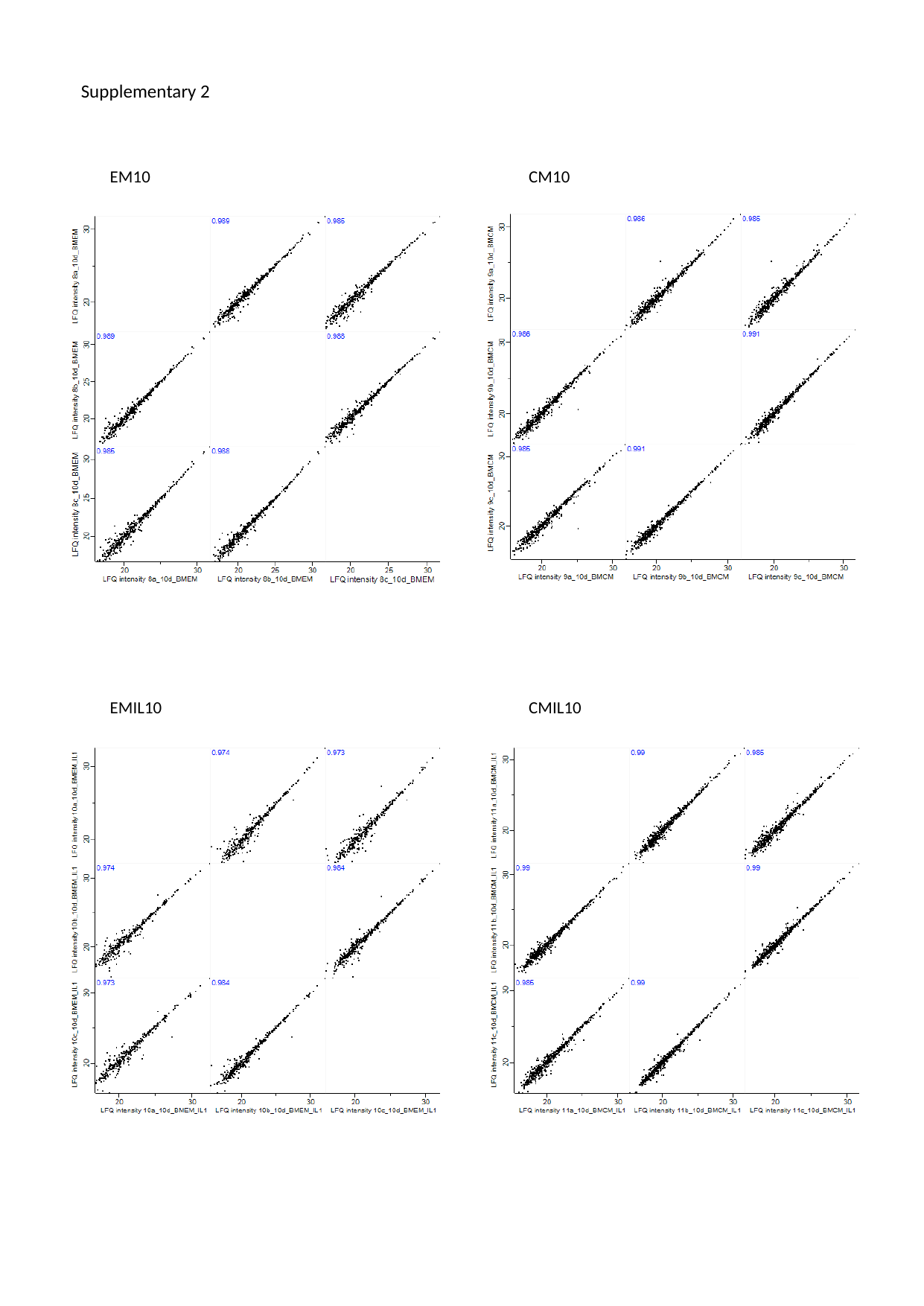

Supplementary 2
EM10
CM10
EMIL10
CMIL10

Supplement: Supplementary file 1 — Additional file 1: Supplementary 1 and 2. Scatter plot with log2-transformed label free quantification (LFQ) values from mass spectrometry analysis of the secretomes harvested after 1) 48 h (48) and 2) 10 days (10) from equine bone marrow-derived mesenchymal stromal cells subjected to IL-1β stimulation (IL) for five days followed by five days without inflammation. At both time points, the secretome from naïve cells (EM) and chondrogenic differentiating cells (CM) was assessed. [file 13287_2020_1706_MOESM1_ESM.zip › Supp_2.pptx]
